# Identification and functional analysis of the geranylgeranyl pyrophosphate synthase gene (*crtE*) and phytoene synthase gene (*crtB*) for carotenoid biosynthesis in *Euglena gracilis*

**DOI:** 10.1186/s12870-015-0698-8

**Published:** 2016-01-05

**Authors:** Shota Kato, Shinichi Takaichi, Takahiro Ishikawa, Masashi Asahina, Senji Takahashi, Tomoko Shinomura

**Affiliations:** Department of Biosciences, School of Science and Engineering, Teikyo University, 1-1 Toyosatodai, Utsunomiya, Tochigi 320-8551 Japan; Department of Biology, Nippon Medical School, 1-7-1 Kyonan-cho, Musashino, Tokyo 180-0023 Japan; Department of Life Science and Biotechnology, Faculty of Life and Environmental Science, Shimane University, 1060 Nishikawatsu, Matsue, Shimane 690-8504 Japan; Plant Molecular and Cellular Biology Laboratory, Department of Biosciences, School of Science and Engineering, Teikyo University, 1-1 Toyosatodai, Utsunomiya, Tochigi 320-8551 Japan

**Keywords:** *Euglena gracilis*, Light stress, Carotenoid biosynthesis, Geranylgeranyl pyrophosphate synthase, CrtE, Phytoene synthase, CrtB

## Abstract

**Background:**

*Euglena gracilis*, a unicellular phytoflagellate within Euglenida, has attracted much attention as a potential feedstock for renewable energy production. In outdoor open-pond cultivation for biofuel production, excess direct sunlight can inhibit photosynthesis in this alga and decrease its productivity. Carotenoids play important roles in light harvesting during photosynthesis and offer photoprotection for certain non-photosynthetic and photosynthetic organisms including cyanobacteria, algae, and higher plants. Although, Euglenida contains β-carotene and xanthophylls (such as zeaxanthin, diatoxanthin, diadinoxanthin and 9′-*cis* neoxanthin), the pathway of carotenoid biosynthesis has not been elucidated.

**Results:**

To clarify the carotenoid biosynthetic pathway in *E. gracilis*, we searched for the putative *E. gracilis* geranylgeranyl pyrophosphate (GGPP) synthase gene (*crtE*) and phytoene synthase gene (*crtB*) by tblastn searches from RNA-seq data and obtained their cDNAs. Complementation experiments in *Escherichia coli* with carotenoid biosynthetic genes of *Pantoea ananatis* showed that *E. gracilis crtE* (*EgcrtE*) and *EgcrtB* cDNAs encode GGPP synthase and phytoene synthase, respectively. Phylogenetic analyses indicated that the predicted proteins of *EgcrtE* and *EgcrtB* belong to a clade distinct from a group of GGPP synthase and phytoene synthase proteins, respectively, of algae and higher plants.

In addition, we investigated the effects of light stress on the expression of *crtE* and *crtB* in *E. gracilis.* Continuous illumination at 460 or 920 μmol m^−2^ s^−1^ at 25 °C decreased the *E. gracilis* cell concentration by 28–40 % and 13–91 %, respectively, relative to the control light intensity (55 μmol m^−2^ s^−1^). When grown under continuous light at 920 μmol m^−2^ s^−1^, the algal cells turned reddish-orange and showed a 1.3-fold increase in the *crtB* expression. In contrast, *EgcrtE* expression was not significantly affected by the light-stress treatments examined.

**Conclusions:**

We identified genes encoding CrtE and CrtB in *E. gracilis* and found that their protein products catalyze the early steps of carotenoid biosynthesis. Further, we found that the response of the carotenoid biosynthetic pathway to light stress in *E. gracilis* is controlled, at least in part, by the level of *crtB* transcription. This is the first functional analysis of *crtE* and *crtB* in *Euglena*.

**Electronic supplementary material:**

The online version of this article (doi:10.1186/s12870-015-0698-8) contains supplementary material, which is available to authorized users.

## Background

*Euglena gracilis*, a eukaryotic unicellular phytoflagellate within Euglenida, is a secondary plant [1] in which the chloroplasts carry chlorophylls *a* and *b* and carotenoids, similar to what is observed in green algae (Chlorophyta) and higher plants [2]. This alga has attracted much attention as a potential feedstock for renewable energy production. In outdoor open-pond cultivation for biofuel production, the productivity of this alga depends on several environmental factors such as light intensity and temperature. Excess direct sunlight can inhibit photosynthesis in this alga and decrease its productivity.

Carotenoids play important roles in photosynthesis and photoprotection of photosynthetic organisms and certain non-photosynthetic organisms. More than 750 natural carotenoids have been isolated from various organisms. Carotenoids are synthesized by phototrophs and non-phototrophs including bacteria, archaea, fungi, algae, and higher plants [3]. In photosynthetic pathways, both carotenoids and chlorophylls constitute light-harvesting pigment-protein complexes in chloroplast membranes. Carotenoids also play important roles in the stabilization of thylakoid membranes [4], in photoprotection (i.e. non-photochemical quenching, the xanthophyll cycle, and scavenging reactive oxygen species) [5], and in the synthesis of abscisic acid [6] and strigolactones [7].

Carotenoids are classified into two classes, carotenes (hydrocarbons) and xanthophylls (oxygenated derivatives of carotenes). Geranylgeranyl pyrophosphate (GGPP; C_20_), the precursor of carotenes, is synthesized from farnesyl pyrophosphate (C_15_) and isopentenyl pyrophosphate (C_5_) by geranylgeranyl pyrophosphate synthase (CrtE, also known as GGPPS or GGPS). Then phytoene (C_40_), the first carotene, is synthesized by the condensation of two molecules of GGPP by phytoene synthase (CrtB, also called Psy or Pys). Subsequently, phytoene is converted into lycopene through desaturation steps and isomerization catalyzed by phytoene desaturase (CrtP, also called Pds), ζ-carotene desaturase (CrtQ, also called Zds) and *cis*-carotene isomerase (CrtH, also called CrtISO) in oxygenic phototrophs. Bicyclic carotenes, α-carotene and β-carotene and their oxygenated derivatives (xanthophylls), are synthesized from lycopene [3, 8].

The distribution of carotenoid species in algae including cyanobacteria, red algae, brown algae, and green algae, has been summarized [8] and suggests that algae have several carotenoid biosynthetic pathways in common with higher plants based on similarities among carotenoid chemical structures. The genes whose products catalyze the early steps of the carotenoid biosynthetic pathways in common with higher plants have been functionally identified in several eukaryotic algae such as *Pyropia umbilicalis* (*ggps*), *Chlamydomonas reinhardtii* (*crtB*), *Haematococcus pluvialis* (*pys*), and *Chlorella zofingiensis* (*psy* and *crtP*) and as well as cyanobacteria such as *Thermosynechococcus elongatus* (*crtE*), *Gloeobacter violaceus* PCC 7421 (*crtB*), *Synechococcus elongatus* PCC 7942 (*pys*), and *Synechocystis* sp. PCC 6803 (*crtQ* and *crtH*) [8–10].

Euglenida contains β-carotene and xanthophylls such as zeaxanthin, diatoxanthin, diadinoxanthin and 9′-*cis* neoxanthin [8, 11–13], however, the biosynthetic pathways and the corresponding genes of carotenoid synthesis in this alga have not been elucidated. In the present study, to clarify the carotenoid biosynthetic pathway of *E. gracilis* within Euglenida, we searched for the orthologs of the GGPP synthase gene and phytoene synthase gene from a series of *E. gracilis* cDNA sequences (Yoshida *et al*., unpublished observations) using tblastn, and we identified *E. gracilis crtE* (*EgcrtE*) and *EgcrtB* encoding GGPP synthase and phytoene synthase, respectively. Phylogenetic analyses indicated that *E. gracilis* CrtE and CrtB belong to a clade that is distinct from groups of algae and higher plants, respectively. In addition, we investigated the effects of light stress on the expression of *crtE* and *crtB* in *E. gracilis*, and revealed that the carotenoid biosynthetic pathway in *E. gracilis* responded to excess light stress at the level of *crtB* transcription.

## Results

### Cloning of *EgcrtE* and *EgcrtB*

We performed BLAST (tblastn) searches against a series of *Euglena* full-length cDNA sequences (Yoshida *et al*., unpublished observations) using *Capsicum annuum* GGPS [GenBank: CAA56554] and *C. annuum* PSY1 [GenBank: CAA48155] as queries. We obtained cDNA sequences of the putative GGPP synthase gene (*crtE*) and phytoene synthase gene (*crtB*) in *E. gracilis*. The cDNA sequences that encode *EgcrtE* and *EgcrtB* from the RNA-seq data each contained a spliced-leader (SL) sequence 5′-TTTTTTTTCG-3′, a characteristic sequence transferred to the 5′ extremity of mRNAs by *trans*-splicing [14]. The presence of SL sequences at the 5′ ends of the cDNAs corresponding to *EgcrtE* and *EgcrtB* indicated that the obtained sequences code for the full-length cDNA. The cDNAs for putative *EgcrtE* and *EgcrtB* (Additional files 1 and 2) were isolated from *E. gracilis* by RT-PCR with primers designed according to the RNA-seq data. The sequences of *EgcrtE* and *EgcrtB* cDNA were submitted to the DDBJ under accession numbers LC062706 and LC062707, respectively.

**Fig. 1:**
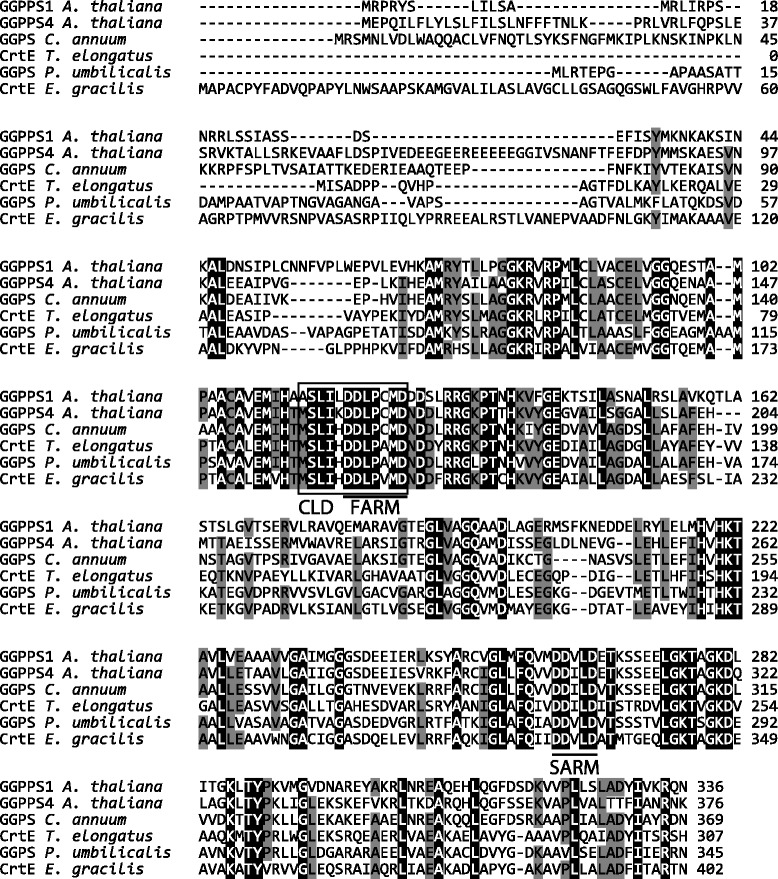
Alignment of the deduced *E. gracilis* CrtE amino acid sequence with known GGPP synthases. The accession numbers are *Arabidopsis thaliana* GGPPS1, [GenBank: NP_175376]; GGPPS4 [GenBank: NP_179960]; *Capsicum annuum* GGPS, [GenBank: CAA56554] and *Thermosynechococcus elongatus* BP-1 CrtE, [GenBank: NP_680811]. Sequence data for GGPS of *Pyropia umbilicalis* [*P_umbilicalis_esContig5139*] was obtained from *Nori*BLAST [58]. Underlined sequences indicate the first and second aspartate-rich motifs, FARM and SARM, respectively. The boxed residues comprise the chain-length determination (CLD) region. Multiple sequence alignment was conducted with Clustal W using MEGA version 6.0 [59]

**Fig. 2:**
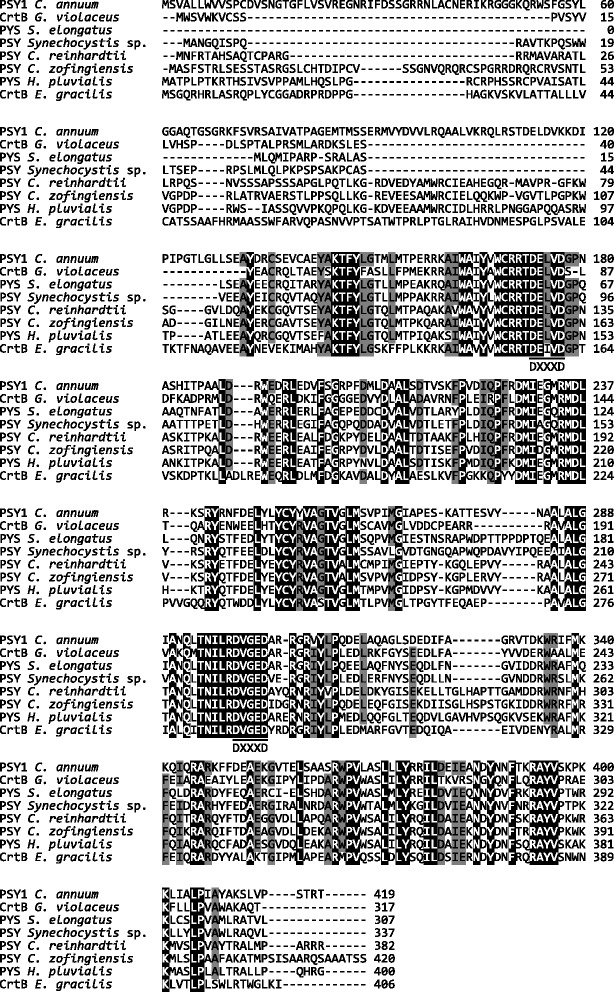
Alignment of the deduced *E. gracilis* CrtB amino acid sequence with known phytoene synthases. The accession numbers are *Capsicum annuum* PSY1, [GenBank: CAA48155]; *Gloeobacter violaceus* PCC 7421 CrtB [GenBank: BAC89685]; *Synechococcus elongatus* PCC 7942 PYS [GenBank: CAA45350]; *Synechocystis* sp. PCC 6803 PYS [GenBank: CAA48922]; *Chlamydomonas reinhardtii* PSY [GenBank: XP_001701192]; *Chlorella zofingiensis* PSY [GenBank: CBW37867] and *Haematococcus pluvialis* PYS [GenBank: AAY53806]. Underlined sequences indicate the two aspartate-rich motifs (DXXXD). Multiple sequence alignment was conducted with Clustal W using MEGA version 6.0 [59]

The first ATG downstream of the SL sequence in both *EgcrtE* and *EgcrtB* cDNA was considered the start codon of the respective mRNA. The deduced amino acid sequences of *EgcrtE* and *EgcrtB* are predicted to be 402 and 406 amino acids in length, respectively (Figs. 1 and 2, and Additional files 1 and 2). The typical signal motif for plastid-targeted proteins in *E. gracilis* [15] was not found in either EgCrtE or EgCrtB with the TMHMM program [16]. Furthermore, no characteristic signal motif was predicted in EgCrtE and EgCrtB with the TargetP program [17].

In the phylogenetic tree for GGPP synthases (Additional file 3), the predicted protein encoded by *EgcrtE* is relatively close to an algal clade including Cyanophyta and Rhodophyta. The amino acid sequence of *E. gracilis* CrtE is 46 and 44 % identical to GGPP synthases of *T. elongatus* and *P. umbilicalis*, respectively, and the corresponding sequence similarities are 59 and 55 %, as aligned with Needle in EMBOSS [18]. EgCrtE contains the typical aspartate-rich motifs conserved in type II GGPPS of eubacteria and plants, namely the first aspartate-rich motif (FARM: DDXXXD) in the chain-length determination (CLD) region, and the second aspartate-rich motif (SARM: DDXXD) [19, 20] (Fig. 1). In the phylogenetic tree (Additional file 4), EgCrtB is in a distinct clade apart from clades of phytoene synthases of cyanobacteria (Cyanophyta) and green algae (Chlorophyta). The deduced amino acid sequence for EgCrtB is 38, 39 and 40 % identical with phytoene synthases of *H. pluvialis*, *C. zofingiensis*, and *C. reinhardtii*, respectively, and the corresponding sequence similarities are 52, 53 and 56 %, as aligned with Needle in EMBOSS [18]. EgCrtB contains two aspartate-rich motifs (DXXXD) conserved among phytoene synthases [21] (Fig. 2).

### Functional analysis of *EgcrtE* and *EgcrtB*

**Fig. 3:**
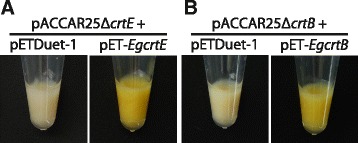
Color complementation experiments in *E. coli* with the *P. ananatis* carotenoid synthetic gene cluster. **a** *E. coli* carrying pACCAR25Δ*crtE* [22] with pETDuet-1 (vector control) or pET-*EgcrtE*. **b** *E. coli* cells carrying pACCAR25Δ*crtB* [23] with pETDuet-1 (vector control) or pET-*EgcrtB. E. coli* strain BL21(DE3) was used as the host. Data are representative of at least eight *E. coli* transformants with similar results

The function of isolated *EgcrtE* and *EgcrtB* cDNA was analyzed with color complementation studies in *Escherichia coli* carrying the carotenoid biosynthetic gene cluster of *Pantoea ananatis* (formerly *Erwinia uredovora*) [22]. *E. coli* transformed with pET-*EgcrtE* and pACCAR25Δ*crtE* [22], which carries *P. ananatis* carotenoid biosynthetic gene cluster (*crtB*, *crtI*; phytoene desaturase, *crtY*; lycopene cyclase, *crtZ*; β-carotene hydroxylase and *crtX*; zeaxanthin glucosidase, but missing *crtE*), showed accumulation of yellow-orange pigments (Fig. 3a). In contrast, this pigmentation was not observed in *E. coli* carrying pACCAR25Δ*crtE* and pETDuet-1 (vector control). In the same way, the function of *EgcrtB* was analyzed in *E. coli* with pACCAR25Δ*crtB* [23] carrying *P. ananatis crtE*, *crtI*, *crtY*, *crtZ* and *crtX*, but missing *crtB. E. coli* co-transformed with pET-*EgcrtB* and pACCAR25Δ*crtB* showed the yellow-orange color (Fig. 3b). These results suggested that the proteins predicted to be encoded by *EgcrtE* and *EgcrtB* have GGPP synthase and phytoene synthase activity, respectively.

**Fig. 4:**
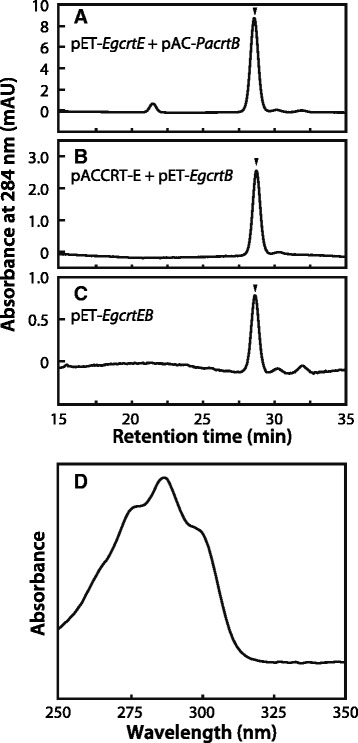
Analysis of phytoene production in *E. coli* by HPLC. HPLC chromatogram (284 nm) of extracts from *E. coli* carrying **a** pET-*EgcrtE* with pAC-*PacrtB*, **b** pACCRT-E [23] with pET-*EgcrtB* and **c** pET-*EgcrtEB*. **d** Absorbance spectrum of phytoene detected at a retention time of 28.6 min. Phytoene was extracted from *E. coli* transformants and analyzed with HPLC in accordance with the method of Takaichi [57]. The arrowheads in the chromatograms indicate the position of phytoene elution. Data are representative of three or four experiments with similar results

The ability of EgCrtE and EgCrtB to function in phytoene production was also investigated by high-performance liquid chromatography (HPLC). Phytoene was detected in *E. coli* harboring *crtE* of *E. gracilis* (pET-*EgcrtE*) and *crtB* of *P. ananatis* (pAC-*PacrtB*) with a retention time of 28.6 min (Fig. 4a, d). Similarly, phytoene production was also observed in *E. coli* carrying *crtE* of *P. ananatis* (pACCRT-E plasmid [23]) and *crtB* of *E. gracilis* (pET-*EgcrtB*) (Fig. 4b). In addition, *E. coli* transformed with pET-*EgcrtEB* carrying *EgcrtE* and *EgcrtB* synthesized phytoene (Fig. 4c). In contrast, phytoene was not detected in *E. coli* carrying either *EgcrtE* or *EgcrtB* alone (Additional file 5A and B). Furthermore, phytoene production was not observed in *E. coli* carrying pAC-*PacrtB* or pACCRT-E with pETDuet-1 (vector control) (Additional file 5C and D). Taken together, these findings indicate that the *crtE* and *crtB* cDNAs isolated from *E. gracilis* code for the GGPP synthase and the phytoene synthase, respectively.

### *crtE* and *crtB* expression in *E. gracilis* in response to light stress

**Fig. 5:**
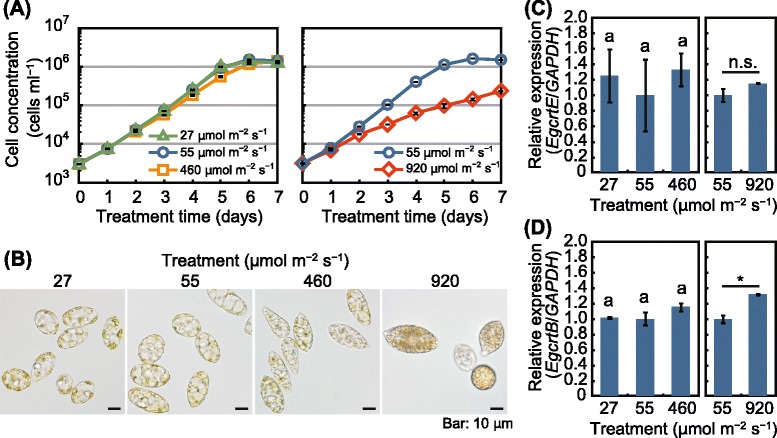
Effects of light intensity on *crtE* and *crtB* expression levels in *E. gracilis*. **a** Time-course of cell concentration of *E. gracilis* grown under continuous light at 27, 55, 460, and 920 μmol m^−2^ s^−1^ at 25 °C. **b** Cells of the alga cultured under the indicated light-stress treatments for 7 days. **c** and **d** Expression levels of *EgcrtE* (**c**) and *EgcrtB* (**d**) in the algal cells treated with the 7-day light-stress treatments. Data are the mean ± SE (*n* = 3). Data are representative of at least two individual experiments with similar results. Bars labeled with the same letter are not significantly different (Tukey’s multiple range test, *P* < 0.05). n.s., not significant; *P < 0.05, *t*-test

Figure 5a shows a time course of *E. gracilis* cell concentration grown under various light intensities. When the cells were grown under continuous light at 55 μmol m^−2^ s^−1^ (control) for 7 days, the cell concentration increased from 3 × 10^3^ cells ml^−1^ to 1.4 − 1.5 × 10^6^ cells ml^−1^. Illumination at 27 μmol m^−2^ s^−1^ did not affect the cell concentration compared with the control throughout the cultivation period. In contrast, a significant decrease in the cell concentration was observed in the algae grown under illumination at 460 and 920 μmol m^−2^ s^−1^ (Fig. 5a). The treatment with light intensity at 460 μmol m^−2^ s^−1^ significantly (*P* < 0.05) decreased the cell concentration to 72, 60, and 77 % of the control after 4, 5, and 6 days of cultivation, respectively. Illumination at 920 μmol m^−2^ s^−1^ decreased the cell concentration to 87 % of the control 1 day after the cultivation, and the degree of inhibition of cell growth increased in a time-dependent manner. After 6 days of cultivation, the concentration of cells illuminated at 920 μmol m^−2^ s^−1^ was decreased to 9 % (1.5 × 10^5^ cells ml^−1^) of the control. After 7 days of treatment at 460 and 920 μmol m^−2^ s^−1^, the cell concentration reached 1.4 × 10^6^ cells ml^−1^ (99 % of control) and 2.4 × 10^5^ cells ml^−1^ (16 % of control), respectively.

Compared with the control, no remarkable difference was observed in the appearance of the algal cells grown under continuous light at 27 μmol m^−2^ s^−1^ for 7 days (Fig. 5b). The cells subjected to the control (55 μmol m^−2^ s^−1^) and to 27 μmol m^−2^ s^−1^ contained translucent granules thought to be paramylon. The translucent granules were also observed in the cells illuminated at 460 μmol m^−2^ s^−1^ for 7 days, although grayish-colored granules (1–2 μm in diameter) also appeared in the cells (Fig. 5b). The cells illuminated at 920 μmol m^−2^ s^−1^ possessed more grayish granules than the cells illuminated at 460 μmol m^−2^ s^−1^. Furthermore, grown under illumination at 920 μmol m^−2^ s^−1^, the cells looked more reddish-orange than the control.

The expression of *crtE* mRNA in *E. gracilis* was not significantly affected by the various light intensities examined when the cells were cultured at 25 °C under continuous illumination (Fig. 5c). In contrast, the expression of *crtB* in the cells illuminated at 920 μmol m^−2^ s^−1^ increased 1.3-fold relative to the control (Fig. 5d). These results indicate that the response of the carotenoid biosynthetic pathway to light stress in *E. gracilis* is controlled, at least in part, at the level of *crtB* transcription.

## Discussion

### Identification of *EgcrtE* and *EgcrtB*

The GGPS of *C. annuum* [24] and the majority of the GGPP synthase family proteins of *Arabidopsis thaliana* [20] localize to plastids. Higher plants have two isoprenoid biosynthetic pathways, namely the plastidial 1-deoxy-d-xylulose 5-phosphate/2-C-methylerythritol 4-phosphate (DOXP/MEP) pathway and cytosolic acetate/mevalonate (MVA) pathway [25, 26]. Green algae (Chlorophyta) lost the MVA pathway during evolution, and thus these algae depend exclusively on the DOXP/MEP pathway [25, 26]. Higher plants and algae depend on isopentenyl pyrophosphate, which is derived from the DOXP/MEP pathway, for the biosynthesis of GGPP and subsequent synthesis of carotenoids in plastids [25]. *Euglena* is exceptional because it lacks the DOXP/MEP pathway and synthesizes isoprenoids via the MVA pathway [26, 27]. This is consistent with the predicted localization of EgCrtE in the cytosol based on TMHMM [16] and TargetP [17].

Phytoene synthases localize to plastids in *A. thaliana*, *Oryza sativa*, and *Zea mays* [21]. In the present study, however, neither TMHMM nor TargetP predicted a typical plastid transit peptide in the N-terminal region of EgCrtB, although it is difficult to exactly predict the plastid-targeted proteins of *E. gracilis* because the system that traffics proteins to *Euglena*’s plastids, which are surrounded by three membranes [1], differs from that of higher plants [28].

Most flagellate green algae have developed a light-sensitive system, the eyespot apparatus, composed of carotenoid-rich lipid globules inside the chloroplast [29]. Proteomic studies indicate that some of the β-carotene biosynthesis enzymes are localized in the eyespot apparatus of *C. reinhardtii* [30] and in β-carotene plastoglobuli in *Dunaliella bardawil* [31], suggesting that part of the β-carotene synthesis occurs in the eyespot globules. *E. gracilis* also possesses an eyespot apparatus (stigma) that contains carotenoids [32], although stigmata of this alga are located in the cytoplasm near the base of the major flagellum [33]. In addition, Kivic and Vesk [33] reported that the stigma of this alga is surrounded by a single membrane and has no structural similarity to the chloroplast. This suggests that EgCrtB might be transported to stigmata as well as plastids and that EgCrtB might contain an as-yet unidentified signal sequence.

Although chloroplasts in *E. gracilis* contain chlorophylls *a* and *b* [2], EgCrtB belongs to a distinct clade apart from groups of green algae (Chlorophyta) and higher plants (Plantae) in the phylogenetic tree (Additional files 3 and 4). This result is consistent with taxonomic relations. *E. gracilis* belongs to Euglenida within supergroup Excavata [34]. Euglenida is a primitive organism that has a common ancestor with *Trypanosoma* sp. (Kinetoplastea) [34–36]. Evolutionarily, Euglenozoa including Euglenida and Kinetoplastea is considered to have branched early from other eukaryotes carrying the symbiont, Chlorophyta [37, 38]. The phylogenetic relationships of GGPP synthase and phytoene synthase proteins among various photoautotrophs (Additional files 3 and 4) might reflect the distinctive evolutionary history of *E. gracilis*.

### *crtE* and *crtB* expression in *E. gracilis* in response to light stress

Steinbrenner and Linden [39] reported that the highest growth rate of *H. pluvialis* is observed under continuous light at 50–150 μmol m^−2^ s^−1^, and illumination at 250 μmol m^−2^ s^−1^ reduces the cell number. Similarly, Wahidin *et al*. [40] showed that the cell concentration of *Nannochloropsis* sp. decreases under illumination at 200 μmol m^−2^ s^−1^. In our preliminary experiment, illumination at 240 μmol m^−2^ s^−1^ had no significant effect on cell concentration throughout the cultivation period compared with the control (data not shown). Illumination at an intensity of ~460 μmol m^−2^ s^−1^ is considered to be a threshold of excess light stress to *E. gracilis* grown under continuous light at 25 °C, and this level of illumination might begin to cause photoinhibition of photosynthesis in this alga. The cell growth delay caused by illumination at 460 μmol m^−2^ s^−1^ was slightly alleviated at the early stationary phase (6 days after the cultivation), and by the end of the cultivation, the algal cells had increased in number as much as the control (Fig. 5a). This result might be due to the shading effects of the grayish granules that accumulated in the cells (Fig. 5b).

When grown under continuous light at 920 μmol m^−2^ s^−1^, the algal cells turned reddish-orange (Fig. 5b). This result is consistent with previous studies indicating that light-stress induces the accumulation of carotenoids in certain green algae such as *Dunaliella salina* [41], *H. pluvialis* [42], and *C. zofingiensis* [43]. Król *et al*. [41] reported that excess irradiance at 2500 μmol m^−2^ s^−1^ induced a comparable accumulation of carotenoids in *D. salina* cells. Wang *et al*. [44] reported that irradiation of *H. pluvialis* at 350 μmol m^−2^ s^−1^ induced an increase in carotenoids, and that the astaxanthin-accumulating red cells were more resistant to very high irradiance (3000 μmol m^−2^ s^−1^) than green cells.

In higher plants, the regulation of carotenoid biosynthesis has mainly been investigated in the context of seedling de-etiolation and the accompanying burst in carotenoid biosynthesis. Lintig *et al*. [45] reported that the expression of the GGPP synthase gene (*ggps*) in *Sinapsis alba* seedlings remained constant during de-etiolation. This report is consistent with our data showing that *EgcrtE* expression remained relatively constant under the light-stress treatments examined (Fig. 5c). Flux of isoprenoids in the MEP pathway in higher plants is mainly controlled by DOXP synthase [46], DOXP reductoisomerase [47], and hydroxymethylbutenyl diphosphate reductase [48]. These three rate-determining enzymes are upregulated and control the metabolic flux to the carotenoid pathway during de-etiolation of *A. thaliana* [49]. Light-induction of the gene *dxs* encoding DOXP synthase was also reported in *Phaeodactylum tricornutum* (diatom) in the dark–light transition [50].

In contrast to *crtE*, *crtB* expression in *E. gracilis* increased by 1.3-fold in response to intense illumination (920 μmol m^−2^ s^−1^; Fig. 5d). This result is consistent with previous studies of light-regulated carotenoid biosynthetic genes. For example, expression of the phytoene synthase gene (*psy*) of *A. thaliana* is upregulated during seedling de-etiolation, resulting in an accumulation of carotenoids [48, 49, 51]. Rodríguez-Villalón *et al*. [49] reported that PSY is the key driver that increases carotenoid synthesis in etiolated seedlings of *A. thaliana* by controlling the metabolic flux to the carotenoid biosynthesis pathway. Light induction of the phytoene synthase gene has also been observed in algae. Bohne and Linden [52] reported that *C. reinhardtii* showed a fast upregulation of *crtB* with a maximum at 1–2 h after the dark-to-light transition. Steinbrenner and Linden [42] reported that continuous high-intensity light (125 μmol m^−2^ s^−1^) leads to a slight increase in *pys* expression followed by moderate astaxanthin accumulation in *H. pluvialis*. This is consistent with our finding that the carotenoid biosynthesis pathway in *E. gracilis* under light stress is controlled, in part, at the transcriptional level of *EgcrtB* downstream of the branch point for carotenoid, chlorophyll, tocopherol, plastoquinone, and gibberellin biosynthesis in isoprenoid metabolism [19].

## Conclusions

We functionally identified the GGPP synthase gene (*EgcrtE*) and phytoene synthase gene (*EgcrtB*), which catalyze the early steps of the carotenoid biosynthetic pathway, in *E. gracilis* within supergroup Excavata. Phylogenetic analyses of GGPP synthase and phytoene synthase proteins indicated that EgCrtE and EgCrtB, respectively, belong to a clade distinct from groups of algae and higher plants, consistent with taxonomic results. In addition, we have found that the carotenoid biosynthetic pathway in *E. gracilis* responded to excess light stress at the level of *EgcrtB* expression. To the best of our knowledge, this is the first report on the functional analysis of *crtE* and *crtB* in *Euglena*.

## Methods

### Biological materials

*Euglena gracilis* Klebs (strain Z) was cultured in 100 ml of Cramer-Myers medium [53] containing 0.1 % ethanol at an initial cell concentration of 3.0 × 10^3^ cells ml^−1^ in a 300-ml conical flask. Algal cells were grown in an incubator (LH-350SP, NK system) with agitation (90 rpm), and illuminated with fluorescent lamps (FL20S EX-N-HG and FL40S EX-N-HG, NEC Lighting). To clone *EgcrtE* and *EgcrtB*, the algal cells were grown at 25 °C under continuous illumination at 55 μmol m^−2^ s^−1^ for 7 days. To analyze the expression levels of *EgcrtE* and *EgcrtB* gene in *E. gracilis* under light stress, algal cells were grown at 25 °C under continuous illumination at 27, 55 (control), 460, and 920 μmol m^−2^ s^−1^ for 7 days. For illumination at 460 and 920 μmol m^−2^ s^−1^, white LED lamps (LLM0175A, Stanley Electric) were used in combination with the fluorescent lamps. Cell concentration was measured daily by counting with a plankton counter (MPC-200, Matsunami Glass Ind.) under a microscope. At 7 days after the cultivation, algal cells were harvested by centrifugation (1000 × *g*, 2 min), and the collected cells were frozen immediately and stored at −60 °C until the RNA was isolated.

### Cloning of *EgcrtE* and *EgcrtB*

Total RNA was isolated from the algal cells with RNAqueous kit (Ambion) and Plant RNA Isolation Aid (Ambion). First-strand cDNA was synthesized with SuperScript First-Strand Synthesis System for RT-PCR (Invitrogen) from total RNA treated with DNase I (Invitrogen). cDNAs containing *EgcrtE* and *EgcrtB* coding sequences were amplified by RT-PCR with PrimeSTAR GXL DNA Polymerase (Takara Bio). Primers used for RT-PCR were as follows: *EgcrtE*, 5′-TTTCGCTCACACGCACAATG-3′ and 5′-CCCAGCGTACAGAAAAGCTA-3′; *EgcrtB*, 5′-TTCGGTCGCTCCCCTTCCA-3′ and 5′-AGCAGCCGAGTATGATACGA-3′. The amplified fragments were gel-purified (Gel/PCR Extraction kit, FastGene) and sub-cloned into pMD20-T vector with Mighty TA-cloning Reagent Set for PrimeSTAR (Takara Bio) and sequenced. *E. coli* strain JM109 (Takara Bio) was used as a host for the plasmids and grown in LB medium [54] at 37 °C in the dark. Ampicillin (50 μg ml^−1^) was added to the medium as needed.

### Construction of plasmids for complementation experiments

The coding sequence of *EgcrtE* cDNA was amplified with PrimeSTAR GXL DNA Polymerase and the primers 5′-TGAATTCCACACGCACAATGGCC-3′ and 5′-ATAAGCTTCAGTTGGTGCGGGC-3′, which contain *EcoR*I and *Hind*III restriction sites, respectively. The coding sequence of *EgcrtB* cDNA was amplified with primers 5′-CTTCCATATGTCCGGCCAGAG-3′ and 5′-TCTCGAGTAAGATCTTCAAGCCC-3′, which contain *Nde*I and *Xho*I restriction sites, respectively. The amplified fragments were gel-purified and sub-cloned into pMD20-T vector with Mighty TA-cloning Reagent Set for PrimeSTAR. *E. coli* strain JM109 was used as a host for the plasmids and grown as described above.

To construct the pET-*EgcrtE*, the coding sequence for *EgcrtE* was cloned into the *EcoR*I/*Hind*III site (multi cloning site 1, MCS1) of pETDuet-1 vector (Novagen) with Ligation Mighty Mix (Takara Bio). pET-*EgcrtB* plasmid was created by cloning the *EgcrtB* sequence into the *Nde*I/*Xho*I sites (MCS2) of pETDuet-1. pET-*EgcrtEB* was created by cloning *EgcrtE* and *EgcrtB* into the *EcoR*I/*Hind*III site (MCS1) and *Nde*I/*Xho*I site (MCS2) of pETDuet-1, respectively.

pAC*-PacrtB* was constructed as follows. The open reading frame of *P. ananatis crtB* was amplified from pACCAR25Δ*crtE* [22] with primers 5′-GAACATATGGCAGTTGGCTCGA-3′ and 5′-ACCTCGAGCTAGAGCGGGC-3′, which contain *Nde*I and *Xho*I restriction site, respectively, and was then cloned into MCS2 of pACYCDuet-1 (Novagen). Restriction enzymes used in this study were purchased from Takara Bio. *E. coli* strain JM109 was used as a host for the plasmids, and grown as described above. Ampicillin (50 μg ml^−1^) and chloramphenicol (30 μg ml^−1^) were added to the medium as needed.

### Complementation experiments

pACCAR25Δ*crtE*, which carries the *P. ananatis* carotenoid synthetic gene cluster (*crtB*, *crtI*, *crtY*, *crtZ* and *crtX*) with the exception of *crtE* was introduced into *E. coli* strain BL21(DE3) (New England BioLabs). The transformant harboring pACCAR25Δ*crtE* was made competent in accordance with the method of Inoue *et al*. [55] and then was transformed with pET-*EgcrtE*. For the functional analysis of *EgcrtB*, *E. coli* strain BL21(DE3) was transformed with both pET-*EgcrtB* and pACCAR25Δ*crtB* [23] carrying the *P. ananatis* gene cluster for zeaxanthin biosynthesis (*crtE*, *crtI*, *crtY*, *crtZ* and *crtX*) with the exception of *crtB*. The transformed *E. coli* cells were grown in 5 ml of LB medium at 37 °C in the dark until the OD_600_ of the culture medium reached 0.6 − 0.8 and then were cultured at 21 °C for 2 days in the medium with 50 μM of isopropyl-β-d-thiogalactopyranoside (IPTG) [56]. Ampicillin (50 μg ml^−1^) and chloramphenicol (30 μg ml^−1^) were added to the medium as needed. The *E. coli* cells were harvested from the medium by centrifugation (3000 × *g*, 5 min).

### Phytoene extraction from *E. coli* and HPLC analysis

For the functional analysis of *EgcrtE*, *E. coli* strain BL21(DE3) was transformed with both pET-*EgcrtE* and pAC-*PacrtB*. For the functional analysis of *EgcrtB*, *E. coli* was co-transformed with pET-*EgcrtB* and pACCRT-E [23], which carries *P. ananatis crtE. E. coli* carrying pET-*EgcrtEB* was also created. The transformed cells were incubated in 5 ml of LB medium at 37 °C until the OD_600_ of the culture medium reached 0.6 − 0.8 and were then grown in the medium with 50 μM IPTG at 21 °C for 2 days in the dark [56]. The *E. coli* cells were harvested by centrifugation (3000 × *g*, 5 min) and frozen at −60 °C until the pigments extraction. Ampicillin (50 μg ml^−1^) and chloramphenicol (30 μg ml^−1^) were added to the medium as needed.

Pigments were extracted twice from the cells with 2 ml of acetone/methanol (7:2, v/v). After centrifugation, extracts were dried with a rotary evaporator. The pigments were dissolved in a small volume of *n*-hexane and then loaded on a silica gel (Wakogel C-300, Wako) column. The extracts were eluted from the column with 1–2 ml of *n*-hexane, and the *n*-hexane phase was evaporated to dryness with the rotary evaporator. The residue was dissolved in a small volume of ethanol and analyzed with an HPLC system as described below. The extraction procedure was conducted under dim light just before HPLC analysis.

The HPLC system was equipped with PEGASIL ODS SP100 column (6φ × 150 mm, Senshu Scientific Co.). The mobile phase was acetonitrile/methanol/tetrahydrofuran (58:35:7, v/v/v) [57] at a flow rate of 1.0 ml min^−1^. Absorbance spectra (250–350 nm, 1.2-nm resolution) and retention times were recorded with SPD-M20A, Photodiode Array Detector (Shimadzu).

### Real-time quantitative PCR (qPCR) analysis of *EgcrtE* and *EgcrtB* expression

Total RNA was extracted from *E. gracilis* cells using RNAqueous kit and Plant RNA Isolation Aid. First-strand cDNA was synthesized from total RNA with QuantiTect Reverse Transcription kit (Qiagen) and used as the template. qPCR was conducted with Fast SYBR Green Master Mix (Applied Biosystems) on 7500 Fast Real-Time PCR System (Applied Biosystems). *GAPDH* [GenBank: L21903.1] was used as a reference gene for normalization of gene expression levels across samples. Primer sequences were as follows: *GAPDH*, 5′-GGTCTGATGACCACCATCCAT-3′ and 5′-TGAGGGTCCATCGACAGTCTT-3′; *EgcrtE*, 5′-GGTCTGGCGTTCCAAATCAT-3′ and 5′-TCATCCTTACCCGCTGTCTTG-3′; and *EgcrtB*, 5′-CGGAGTGACGGAGGATCAGA-3′ and 5′-ATCAAGGCCCGGTAATTCTCA-3′. qPCR analysis was performed in triplicate on each of three independent samples for each treatment.

## Availability of supporting data

The data sets supporting the results of this article are included within the article and its additional files.

## Additional files

Additional file 1: Figure S1. Nucleotide sequence of *E. gracilis crtE* and its deduced amino acid sequence. (PDF 1127 kb) Additional file 2: Figure S2. Nucleotide sequence of *E. gracilis crtB* and its deduced amino acid sequence. (PDF 1130 kb) Additional file 3: Figure S3. Phylogenetic relationships of the deduced EgCrtE amino acid sequence and known GGPP synthases. Numbers in parentheses are accession numbers of GGPP synthases. Sequence data for GGPS of *Pyropia umbilicalis* [*P_umbilicalis_esContig5139*] was obtained from *Nori*BLAST [58]. The phylogenetic tree was constructed with the neighbor-joining method using MEGA version 6.0 [59]. Bootstrap values from the percentages of 1000 replications are indicated beside each node. (PDF 972 kb) Additional file 4: Figure S4. Phylogenetic relationships of the deduced EgCrtB amino acid sequence and known phytoene synthases. Numbers in parentheses are accession numbers of phytoene synthases. The phylogenetic tree was constructed with the neighbor-joining method using MEGA version 6.0 [59]. Bootstrap values from the percentages of 1000 replications are indicated beside each node. (PDF 988 kb) Additional file 5: Figure S5. Analysis of phytoene production in *E. coli* by HPLC. HPLC chromatogram (284 nm) of extracts from *E. coli* cells carrying (A) pET-*EgcrtE*, (B) pET-*EgcrtB*, (C) pAC-*PacrtB* with pETDuet-1 (vector control), and (D) pACCRT-E [23] with pETDuet-1. Data are representative of three or four experiments with similar results. Phytoene was eluted at 28.6 min (Fig. 4). The peak at 21.5 min was not carotenoid. (PDF 1108 kb)

## Abbreviations

CrtB Psy, Pys: Phytoene synthase
CrtE GGPPS, GGPS: geranylgeranyl pyrophosphate synthase
CrtH CrtISO: *cis*-carotene isomerase
CrtI CrtP: Phytoene desaturase
CrtQ: ζ-carotene desaturase
CrtX: Zeaxanthin glucosidase
CrtY: Lycopene cyclase
CrtZ: β-carotene hydroxylase
DOXP: 1-deoxy-d-xylulose 5-phosphate
GGPP: Geranylgeranyl pyrophosphate
HPLC: High-performance liquid chromatography
IPTG: Isopropyl-β-d-thiogalactopyranoside
MEP: 2-C-methylerythritol 4-phosphate
MEV: Mevalonate
