# Supplementary material for: Identification and functional analysis of the geranylgeranyl pyrophosphate synthase gene (crtE) and phytoene synthase gene (crtB) for carotenoid biosynthesis in Euglena gracilis
Source: BMC Plant Biol. 2016 Jan 5;16:4. doi: 10.1186/s12870-015-0698-8 (PMC4702402; doi:10.1186/s12870-015-0698-8)
Supplement: Additional file 1: Figure S1. — Nucleotide sequence of E. gracilis crtE and its deduced amino acid sequence. (PDF 1127 kb) [file 12870_2015_698_MOESM1_ESM.pdf]

TTTCGCTCACACGCACAATGGCCCGGCCTGCCCATACTTCGCTGACGTGCAGCCCGCGC
M A P A C P Y F A D V Q P A
CATACCTGAACTGGTCTGCGGCGCCGACCAAGGCGATGGGGGTAGCCCTCATCCTGGCGT
P Y L N W S A A P T K A M G V A L I L A
CGCTGGCAGTGGGCTGTCTCCTTGGCAGTGCAGGCCAGGGCAGCTGGCTGTTTGCTGTTG
S L A V G C L L G S A G Q G S W L F A V
GCCATCGGCCGGTGGTGGCTGGCCGGCCTACCCCGATGGTGGTGCGCAGTAACCCCGTGG
G H R P V V A G R P T P M V V R S N P V
CATCCGCCTCCCGGCCCATCATCCAGTTGTACCCACGGCGTGAGGAGGCGTTGAGGTCCA
A S A S R P I I Q L Y P R R E E A L R S
CGCTTGTTGCAAATGAGCCTGTGGCGGCAGACTTCAACCTTGGCAAGTACATCATGGCCA
T L V A N E P V A A D F N L G K Y I M A
AGGCAGCGGCGGTGGAGGCGGCGCTGGACAAGTACGTCCCGAACGGGCTACCCCCACACC
K A A A V E A A L D K Y V P N G L P P H
CGAAGGTCATCTTTGATGCGATGAGACACTCCCTCCTGGCCGGTGGCAAGAGGATCCGGC
P K V I F D A M R H S L L A G G K R I R
CGGCGCTGGTCATCGCGGCGTGCGAGATGGTGGGCGGCACTCAGGAGATGGCGATGCCCA
P A L V I A A C E M V G G T Q E M A M P
CCGCCTGTGCCCTGGAGATGGTCCACACCATGTCCCTCATCCACGACGACCTCCCCGTGA
T A C A L E M V H T M S L I H D D L P V
TGGACAACGACGACTTCAGGCGGGGGAAGCCAACTTGCCACAAGGTGTATGGGGAGGCGA
M D N D D F R R G K P T C H K V Y G E A
TCGCCCTGCTGGCCGGGGATGCGCTGCTCGCGGAGTCGTTCTCGCTGATTGCGAAGGAGA
I A L L A G D A L L A E S F S L I A K E
CGAAGGGGGTCCCAGCGGACCGCGTGTTGAAGTCCATCGCCAACCTGGGCACCCTGGTGG
T K G V P A D R V L K S I A N L G T L V
GCTCCGAGGGATTGGTGGGCGGGCAGGTGATGGACATGGCGTACGAGGGGAAGGGCGACA
G S E G L V G G Q V M D M A Y E G K G D
CCGCCACACTGGAAGCAGTCGAGTACATCCACATCCACAAGACGGCGGCACTGCTGGAGG
T A T L E A V E Y I H I H K T A A L L E
CCGCGGTGTGGAACGGCGCGTGTATCGGCGGGGCGTCGGACCAGGAGCTGGAGGTGCTGC
A A V W N G A C I G G A S D Q E L E V L
GCCGGTTCGCCCAGAAGATCGGTCTGGCGTTCCAAATCATCGACGACGTGCTGGACGCCA
R R F A Q K I G L A F Q I I D D V L D A
CCATGACTGGGGAGCAGCTGGGCAAGACAGCGGGTAAGGATGAGGCGGTGGCCAAGGCCA
T M T G E Q L G K T A G K D E A V A K A
CGTACGTGCGGGTGGTCGGCCTGGAGCAGTCCCGCGCCATCGCCCAGCGGCTCATCGCGG
T Y V R V V G L E Q S R A I A Q R L I A
AGGCGAAGGCGGACCTCGCCCCCTACGGCGCCAAGGCGGTCCCCCTGCTGGCCCTCGCCG
E A K A D L A P Y G A K A V P L L A L A
ACTTCATCACCGCCCGCACCAACTGAGGCTGCCGTCGTCGCCCCCGGGCTAACCCTACCG
D F I T A R T N
CCTCCCAACCAGTCCCCCCCCCACTGCAGAGTCGCTAGGCCCCGAGCTACGTCCCCAGC

CCACCCATTTGCCGGTCTGCCTGGCACCTTGGGTGGCGCGGCGGGGCTTGCACGGGTGTC

GGCCGGTCCCCCTGGTAGACCCGTCCCCGGCCTCGGTAGCTTTTCTGTACGCTGGG
