# Supplementary material for: Identification and functional analysis of the geranylgeranyl pyrophosphate synthase gene (crtE) and phytoene synthase gene (crtB) for carotenoid biosynthesis in Euglena gracilis
Source: BMC Plant Biol. 2016 Jan 5;16:4. doi: 10.1186/s12870-015-0698-8 (PMC4702402; doi:10.1186/s12870-015-0698-8)
Supplement: Additional file 2: Figure S2. — Nucleotide sequence of E. gracilis crtB and its deduced amino acid sequence. (PDF 1130 kb) [file 12870_2015_698_MOESM2_ESM.pdf]

```
TTCGGTCGCTCCCCTTCCACATGTCCGGCCAGAGGCATCGGCTGGCCTCCCGGCAGCCCC
                     M  S  G  Q  R  H  R  L  A  S  R  Q  P
TCTATTGCGGTGGCGCTGACCGCCCCCGGGACCCCCGGGCCACGCTGGCAAGGTGTCCA
L  Y  C  G  G  A  D  R  P  R  D  P  P  G  H  A  G  K  V  S
AGGTGCTGGCCACCACCGCCCTCCTACTGGTCTGTGCCACCTCCAGTGCGGCGTTCCACC
K  V  L  A  T  T  A  L  L  L  V  C  A  T  S  S  A  A  F  H
GCATGGCAGCCTCATCCTGGTTTGCTCGCGTTCAGCCGGCCTCAAACGTGGTCCCTACAA
R  M  A  A  S  S  W  F  A  R  V  Q  P  A  S  N  V  V  P  T
GCGCAACATGGACCCCACGGCTCCCCACTGGCCTGCGGGCCATCCATGTGGACAATATGG
S  A  T  W  T  P  R  L  P  T  G  L  R  A  I  H  V  D  N  M
AGTCGCCGGGGCTACCATCCGTGGCGCTGGAGACGAAGACCTTCAATGCACAGGCCGTGG
E  S  P  G  L  P  S  V  A  L  E  T  K  T  F  N  A  Q  A  V
AGGAGGCCTATAATGAGGTCGAGAAGATTATGGCCCACTATGCCAAAACATTCTATCTGG
E  E  A  Y  N  E  V  E  K  I  M  A  H  Y  A  K  T  F  Y  L
GCTCCAAGTTCTTCCCCCTGAAGAAGCGGAAAGCCATCTGGGCAGTGTACGTGTGGTGCC
G  S  K  F  F  P  L  K  K  R  K  A  I  W  A  V  Y  V  W  C
GTCGCACGGATGAGATTGTGGACGGGCCCACCGTGTCGAAAGATCCGACCAAGCTGCTGG
R  R  T  D  E  I  V  D  G  P  T  V  S  K  D  P  T  K  L  L
CGGACCTGCGGGAGTGGGAGCAGCGCCTCGACCTGATGTTCGATGGGAAGGCGGTGGACG
A  D  L  R  E  W  E  Q  R  L  D  L  M  F  D  G  K  A  V  D
CGCTGGACTACGCCCTGGCCGAGTCGCTGAAGGTCTTCCCGGGGAAGAAGCAGCCGTACT
A  L  D  Y  A  L  A  E  S  L  K  V  F  P  G  K  K  Q  P  Y
ACGACATGATCGAGGGCATGCGGATGGACCTGCCCGTTGTGGGGCAGCAGCGGTACCAAA
Y  D  M  I  E  G  M  R  M  D  L  P  V  V  G  Q  Q  R  Y  Q
CCTGGGACGACCTGTACCTGTACTGCTACCGGGTGGCGTCCACCGTGGGGCTAATGACGC
T  W  D  D  L  Y  L  Y  C  Y  R  V  A  S  T  V  G  L  M  T
TGCCGGTGATGGGGCTCACGCCGGGCTACACCTTCGAGCAGGCGGAGCCGCCCGCGGTGG
L  P  V  M  G  L  T  P  G  Y  T  F  E  Q  A  E  P  P  A  V
CCCTGGGCATCGCCCTGCAGATCACCAACATCCTGCGCGACGTCGGGGAGGACTACCGAG
A  L  G  I  A  L  Q  I  T  N  I  L  R  D  V  G  E  D  Y  R
ATCGTGGCCGGATCTACCTGCCCCTTGAGGACATGGCTCGGTTCGGAGTGACGGAGGATC
D  R  G  R  I  Y  L  P  L  E  D  M  A  R  F  G  V  T  E  D
AGATCCAAGCAGAAATCGTGGATGAGAATTACCGGGCCTTGATGCGATTCGAAATTCAAC
Q  I  Q  A  E  I  V  D  E  N  Y  R  A  L  M  R  F  E  I  Q
GTGCCCGAGACTACTATGCACTGGCCAAAACAGGGATCCCGATGCTGGCTCCGGAGGCCC
R  A  R  D  Y  Y  A  L  A  K  T  G  I  P  M  L  A  P  E  A
GGATGCCGGTGCAGTCGTCGCTGGACCTCTACAGCCAGATCCTGGACTCCATCGAGCGGA
R  M  P  V  Q  S  S  L  D  L  Y  S  Q  I  L  D  S  I  E  R
ACGACTACGACAACTTCCGCCAGCGGGCCTACGTCTCCAACTGGAACAAGCTGGTCACCC
N  D  Y  D  N  F  R  Q  R  A  Y  V  S  N  W  N  K  L  V  T
TCCCCCTCTCCTGGCTCCGTACCTGGGGCTTGAAGATCTGATGCGGGCCCGAGGCAGCCC
L  P  L  S  W  L  R  T  W  G  L  K  I
AAAGATGGAGAGCCGCAAGGAACGGTGTTCAGTTTGCCCCCTGCCGTTTCGTATCATACT

CGGCTGCT
```
