# Supplementary figures and images for: Identification and functional analysis of the geranylgeranyl pyrophosphate synthase gene (crtE) and phytoene synthase gene (crtB) for carotenoid biosynthesis in Euglena gracilis
Source: BMC Plant Biol. 2016 Jan 5;16:4. doi: 10.1186/s12870-015-0698-8 (PMC4702402; doi:10.1186/s12870-015-0698-8)

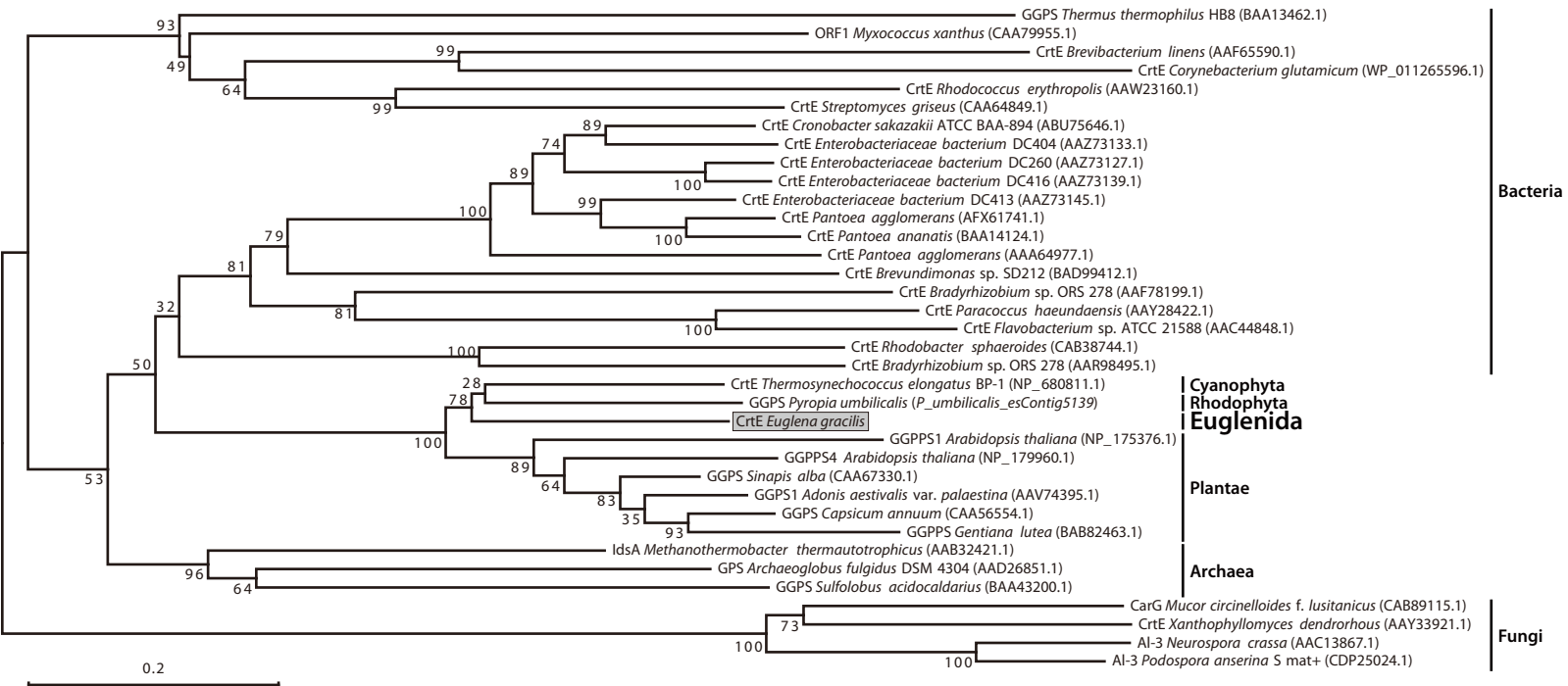

Supplement: Additional file 3: Figure S3. — Phylogenetic relationships of the deduced EgCrtE amino acid sequence and known GGPP synthases. Numbers in parentheses are accession numbers of GGPP synthases. Sequence data for GGPS of Pyropia umbilicalis [P_umbilicalis_esContig5139] was obtained from NoriBLAST [58]. The phylogenetic tree was constructed with the neighbor-joining method using MEGA version 6.0 [59]. Bootstrap values from the percentages of 1000 replications are indicated beside each node. (PDF 972 kb) [file 12870_2015_698_MOESM3_ESM.pdf]

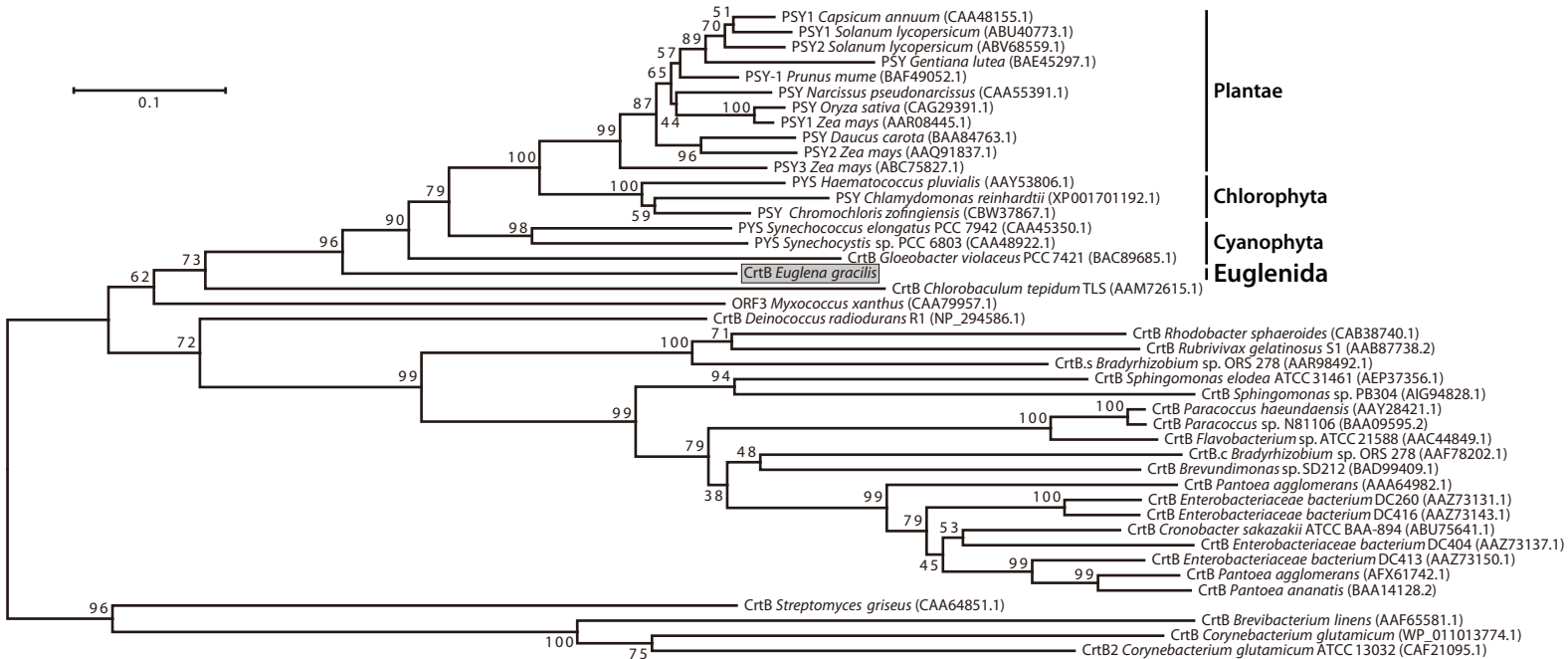

Supplement: Additional file 4: Figure S4. — Phylogenetic relationships of the deduced EgCrtB amino acid sequence and known phytoene synthases. Numbers in parentheses are accession numbers of phytoene synthases. The phylogenetic tree was constructed with the neighbor-joining method using MEGA version 6.0 [59]. Bootstrap values from the percentages of 1000 replications are indicated beside each node. (PDF 988 kb) [file 12870_2015_698_MOESM4_ESM.pdf]

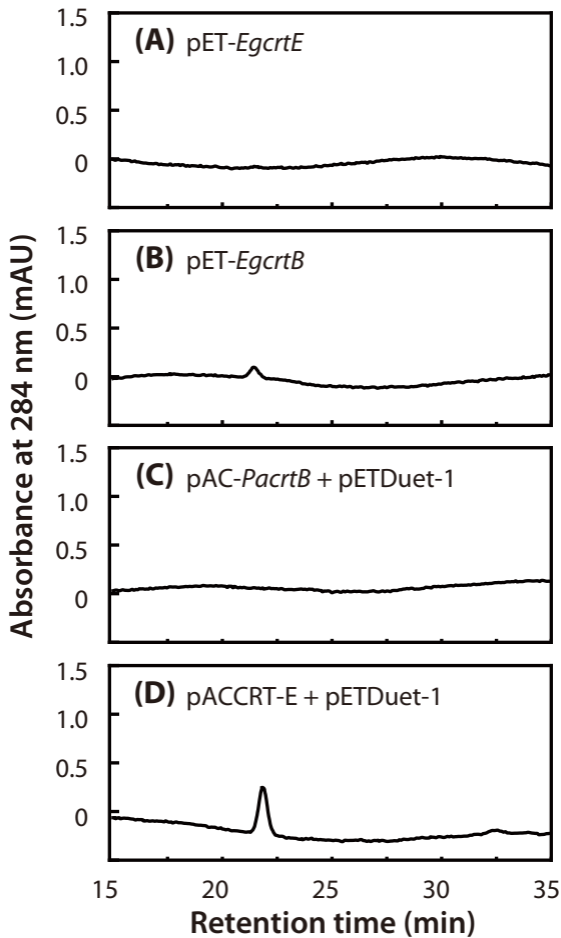

Supplement: Additional file 5: Figure S5. — Analysis of phytoene production in E. coli by HPLC. HPLC chromatogram (284 nm) of extracts from E. coli cells carrying (A) pET-EgcrtE, (B) pET-EgcrtB, (C) pAC-PacrtB with pETDuet-1 (vector control), and (D) pACCRT-E [23] with pETDuet-1. Data are representative of three or four experiments with similar results. Phytoene was eluted at 28.6 min (Fig. 4). The peak at 21.5 min was not carotenoid. (PDF 1108 kb) [file 12870_2015_698_MOESM5_ESM.pdf]
